# Supplementary material for: Endogenous metabolites of vitamin E limit inflammation by targeting 5-lipoxygenase
Source: Nat Commun. 2018 Sep 20;9:3834. doi: 10.1038/s41467-018-06158-5 (PMC6148290; doi:10.1038/s41467-018-06158-5)
Supplement: Supplementary file 1 — Supplementary Information [file 41467_2018_6158_MOESM1_ESM.pdf]

Supplementary information

Endogenous metabolites of vitamin E limit inflammation by targeting 5-lipoxygenase

Pein et al.

Supplementary Figures

Supplementary Fig. 1 | Endogenous metabolites of vitamin E inhibit 5-LO to suppress inflammation.

Supplementary Fig. 2 | Molecular docking simulations.

Supplementary Fig. 3 | Administration of α-T (**1a**) to mice with established peritonitis neither elevates LCM levels in the peritoneal exudate nor in infiltrated peritoneal cells.

Supplementary Fig. 4 | Design of the human liver-on-chip and α-T-13'-COOH (**4a**) biosynthesis.

Supplementary Fig. 5 | δ-TE-13'-COOH (**4h**) preferentially inhibits the formation of 5-LO-derived products within eicosanoid biosynthesis.

Supplementary Fig. 6 | Time-dependent immune cell infiltration into the peritoneal cavity in zymosan-induced murine peritonitis.

Supplementary Fig. 7 | Effect of α-T-13'-COOH (**4a**) on the lipid mediator profiles of peritoneal exudates and plasma during resolution of mouse peritonitis.

Supplementary Fig. 8 | Uncropped Western blots.

Supplementary Fig. 9 | Multi-step strategy for the semi-synthesis of α-TE-13'-CH_2OH (**2e**)

Supplementary Fig. 10 | Multi-step strategy for the semi-synthesis of β-TE-13'-CH_2OH (**2f**)

Supplementary Fig. 11 | Semi-synthesis of methyl 19,20-dihydro-δ-garcinoate

Supplementary Fig. 12 | Semi-synthesis of δ-DE-13'-COOH (**4l**).

Supplementary Fig. 13 | Semi-synthesis of δ-TE-13'-CONHBz (**7**).

Supplementary Tables

Supplementary Table 1 | Docking scores for Ts, TEs, LCMs and vitamin E derivatives

Supplementary Table 2 | Amino acid sequence alignment of human LO isoenzymes

Supplementary Table 3 | Conditions for the quantitation of LCMs by UPLC-MS/MS

Supplementary Table 4 | Plasma and exudate levels of vitamin E metabolites in mouse peritonitis

Supplementary Table 5 | Sequence of wildtype 5-LO and site-directed mutants in the expression vectors 5-LO_wt, 5-LO_3W and 5-LO_Arg101Asp

Supplementary Notes

Supplementary Note 1 | Semi-synthesis of LCMs

Supplementary references

Supplementary Figures

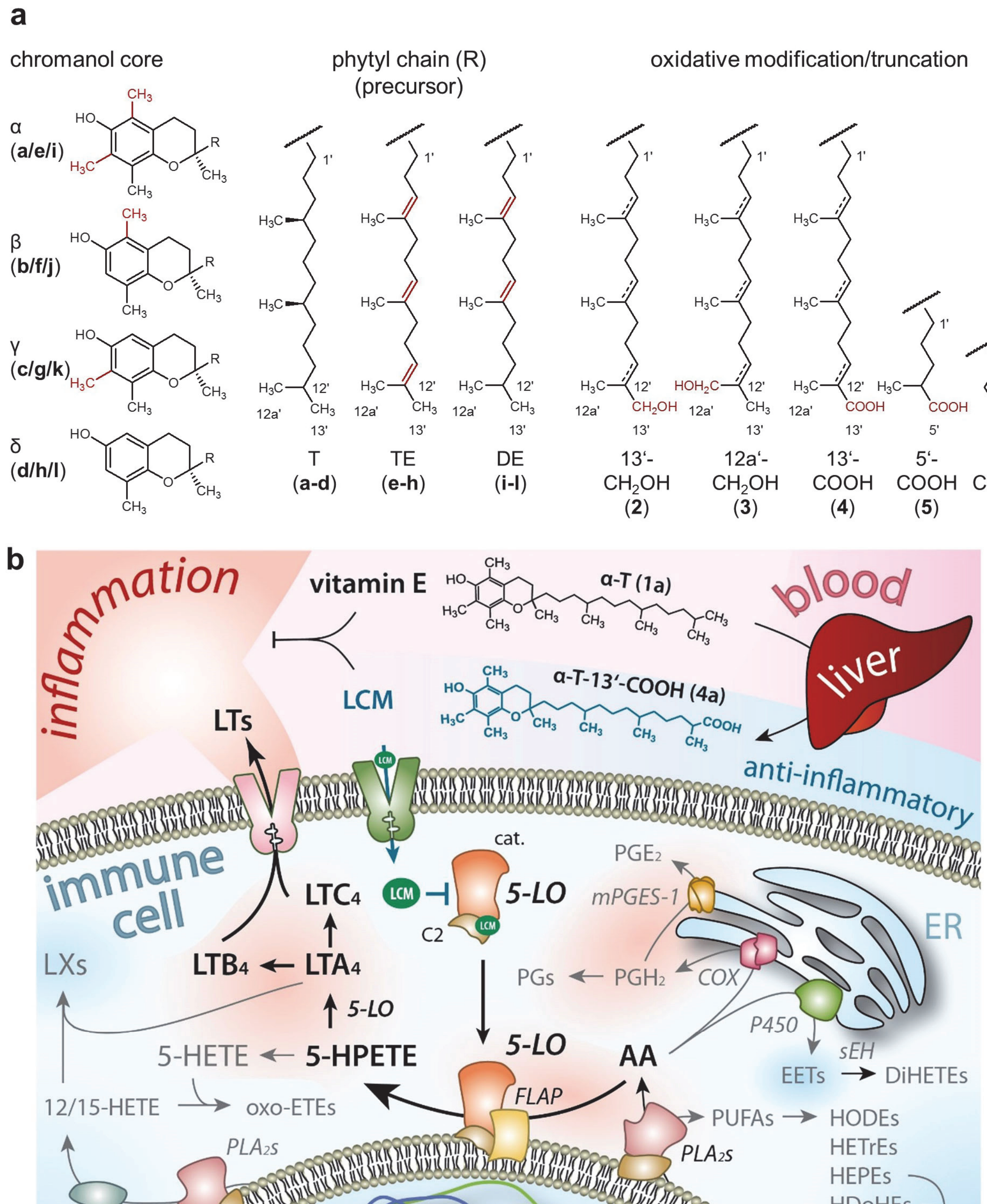

Supplementary Fig. 1 Endogenous metabolites of vitamin E inhibit 5-LO to suppress inflammation. **a** Natural vitamin E forms and metabolites. Abbreviations used: T, tocopherols; TE, tocotrienols; DE, tocodienols. **b** Schematic overview about eicosanoid and docosanoid biosynthesis. The biosynthesis of leukotrienes (LTs) in immune cells is initiated by phospholipases (PL)A_2, which release polyunsaturated fatty acids (PUFAs) such as arachidonic acid (AA) from membrane phospholipids. AA is transferred by 5-LO-activating protein (FLAP) to 5-LO at the nuclear membrane, dioxygenized to 5-hydroperoxy-eicosatetraenoic acid (5-HPETE) and either reduced to 5-hydroxy-eicosatetraenoic acid (5-HETE) or rearranged to LTA_4 by the LTA_4 synthase activity of 5-LO. Conversion of LTA_4 to LTB_4

and cysteinyl-LTs (*i.e.*, LTC_4) yields potent mediators of inflammation. In concert with cyclooxygenase (COX) isoenzymes, cytochrome P450 monooxygenases (P450) and other enzymes, LOs (5-, 12-, 15-LO) produce a large spectrum of pro-inflammatory/non-resolving and anti-inflammatory/pro-resolving lipid mediators. Mode of action of LCMs: α-T (**1a**, vitamin E) is metabolized in the liver to α-T-13'-COOH (**4a**, LCM), which binds to 5-LO as high-affinity inhibitor at the interface of the catalytic (cat.) and regulatory C2-like domains. α-T-13'-COOH (**4a**) is released from the liver and present in plasma at concentrations that are sufficient to inhibit 5-LO, accumulates in immune cells at inflammatory sites and counteracts acute inflammation by suppressing LT formation. Abbreviations used: DiHETE, dihydroxy-eicosatetraenoic acid; EET, epoxy-eicosatrienoic acid; ER, endoplasmic reticulum; HDoHE, hydroxy-docosahexaenoic acid; HEPE, hydroxy-eicosapentaenoic acid; HETrE, hydroxy-eicosatrienoic acid; HODE, hydroxy-octadecadienoic acid; LXs, lipoxins; MaRs, maresins; oxo-ETE, 5-oxo-eicosatetraenoic acid; PDs, protectins; RVs, resolvins; sEH, soluble epoxide hydrolase.

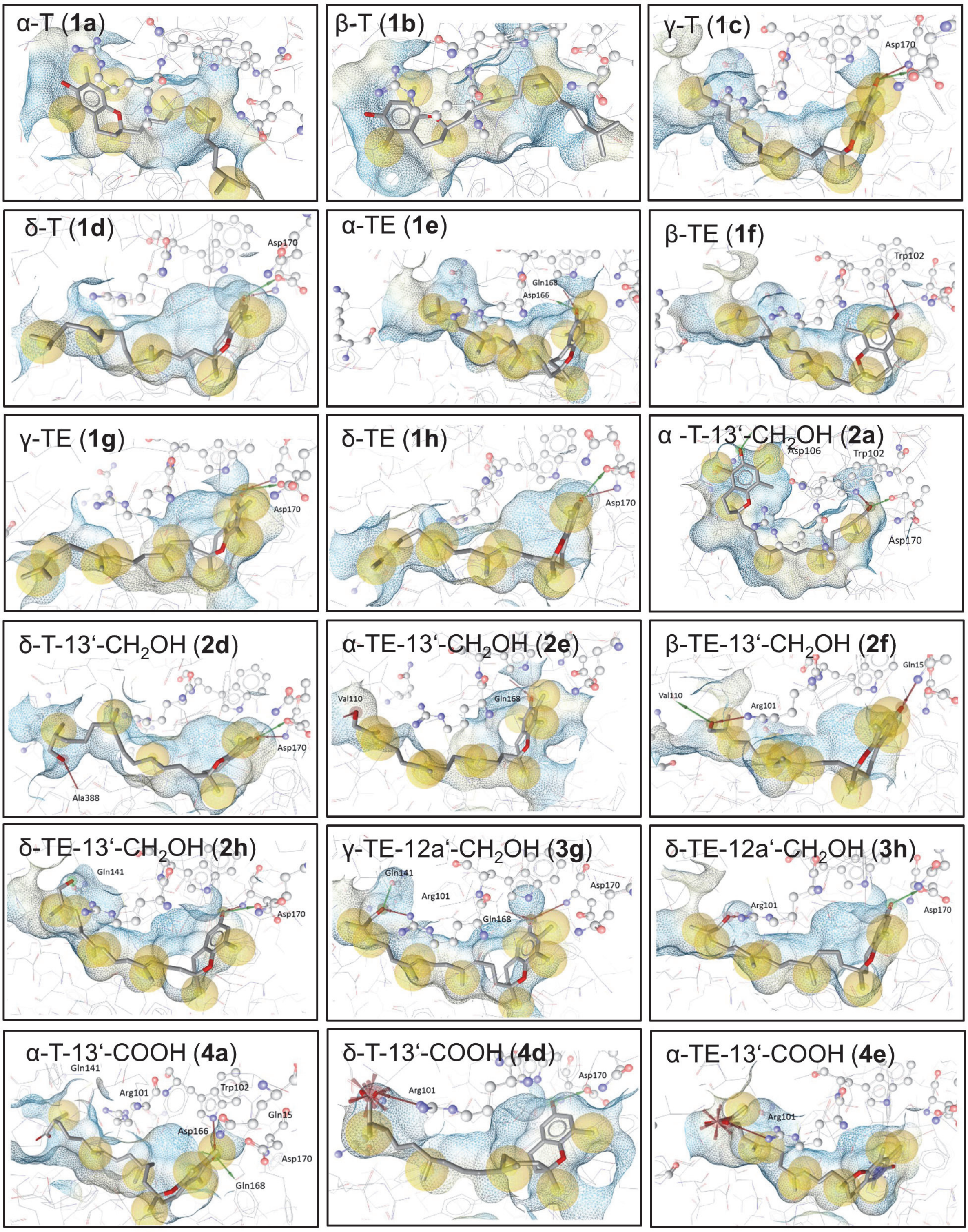

α-T (**1a**)
β-T (**1b**)
γ-T (**1c**)
Asp170
δ-T (**1d**)
Asp170
α-TE (**1e**)
Gln168
Asp166
β-TE (**1f**)
Trp102
γ-TE (**1g**)
Asp170
δ-TE (**1h**)
Asp170
α -T-13‘-CH_2OH (**2a**)
Asp106
Trp102
Asp170
δ-T-13‘-CH_2OH (**2d**)
Asp170
Ala388
α-TE-13‘-CH_2OH (**2e**)
Val110
Gln168
β-TE-13‘-CH_2OH (**2f**)
Val110
Arg101
Gln15
δ-TE-13‘-CH_2OH (**2h**)
Gln141
Asp170
γ-TE-12a‘-CH_2OH (**3g**)
Gln141
Arg101
Gln168
Asp170
δ-TE-12a‘-CH_2OH (**3h**)
Arg101
Asp170
α-T-13‘-COOH (**4a**)
Gln141
Arg101
Trp102
Gln15
Asp166
Asp170
Gln168
δ-T-13‘-COOH (**4d**)
Arg101
Asp170
α-TE-13‘-COOH (**4e**)
Arg101

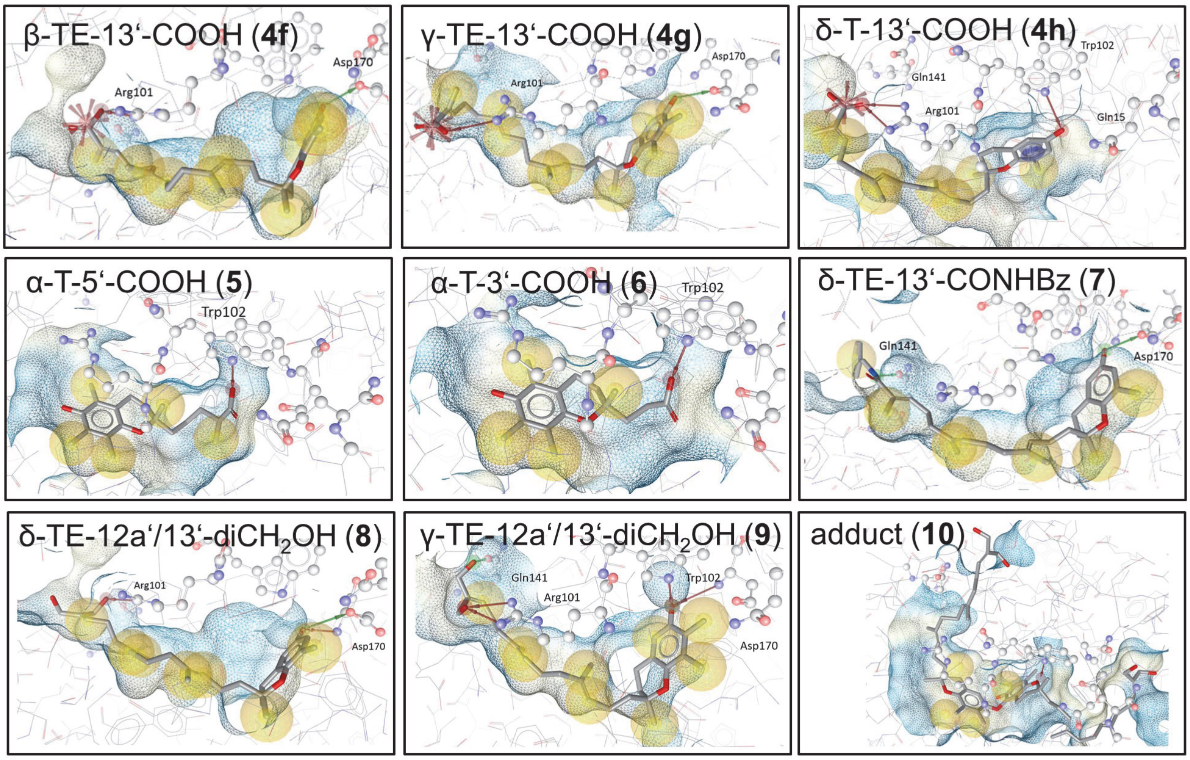

Supplementary Fig. 2 Molecular docking simulations of 5-LO. Simulations show the interaction with Ts, TEs, LCMs, δ-TE-13’-CONHBz (**7**), δ-TE-12a‘/13‘-diCH_2OH (**8**), γ-TE-12a‘/13‘-diCH_2OH (**9**) and the adduct (**10**) at the interface of the catalytic and regulatory C2-like domains.

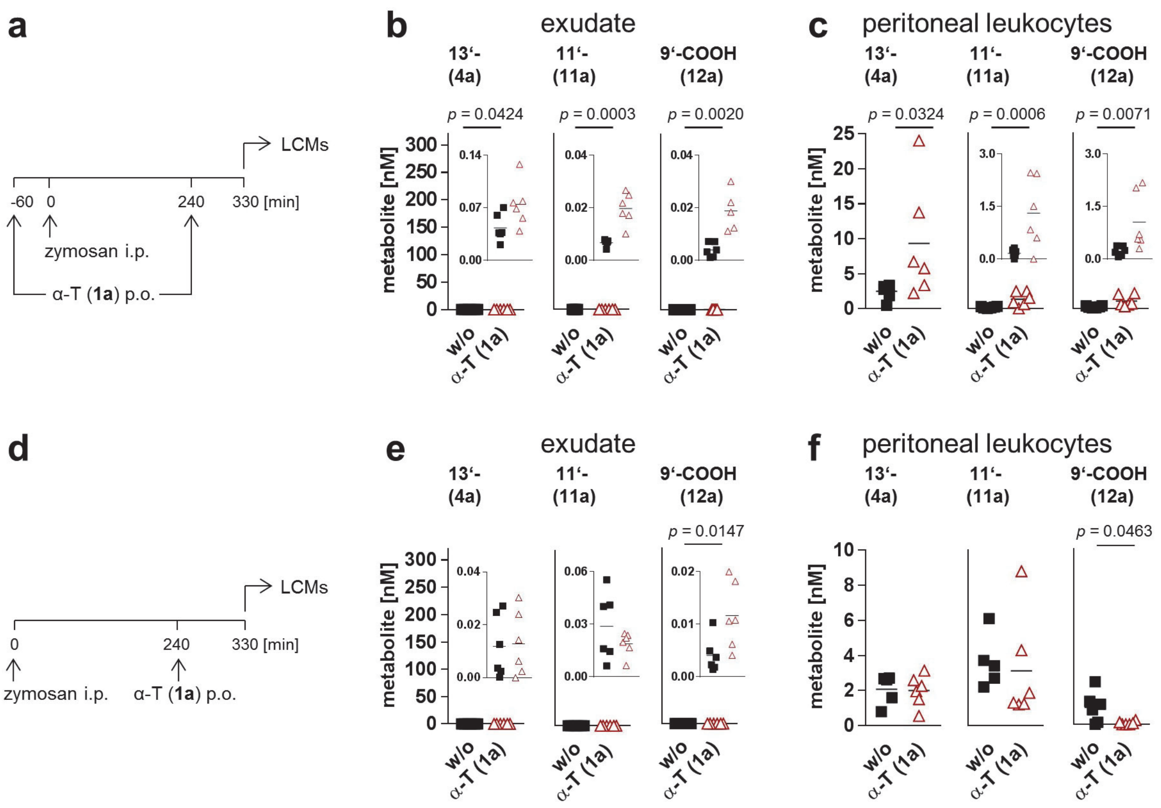

Supplementary Fig. 3 Administration of α-T (**1a**) to mice with established peritonitis neither elevates LCM levels in the peritoneal exudate nor in infiltrated peritoneal cells. **a**, **d** Time-scales for zymosan-induced murine peritonitis. **b**, **c**, **e**, **f** Concentration of α-T-13'-COOH (**4a**), α-T-11'-COOH (**11a**) and α-T-9'-COOH (**12a**) in peritoneal exudates (**b**, **e**) and infiltrated peritoneal cells (**c**, **f**) of mice with acute peritonitis 5.5 h post zymosan injection. α-T (50 mg/kg) was either administered p.o. twice (1 h before plus 4 h after zymosan; **b**, **c**) or once (4 h after zymosan; **e**, **f**). **c**, **f** Average intracellular concentrations were calculated for spherical peritoneal cells with a diameter of 13 µm. Mean with single data from n = 6 mice, except for **b**: w/o, α-T-11'-COOH (**11a**) and α-T (**1a**), α-T-9'-COOH (**12a**) with n = 5 mice. Two-tailed unpaired student *t*-test.

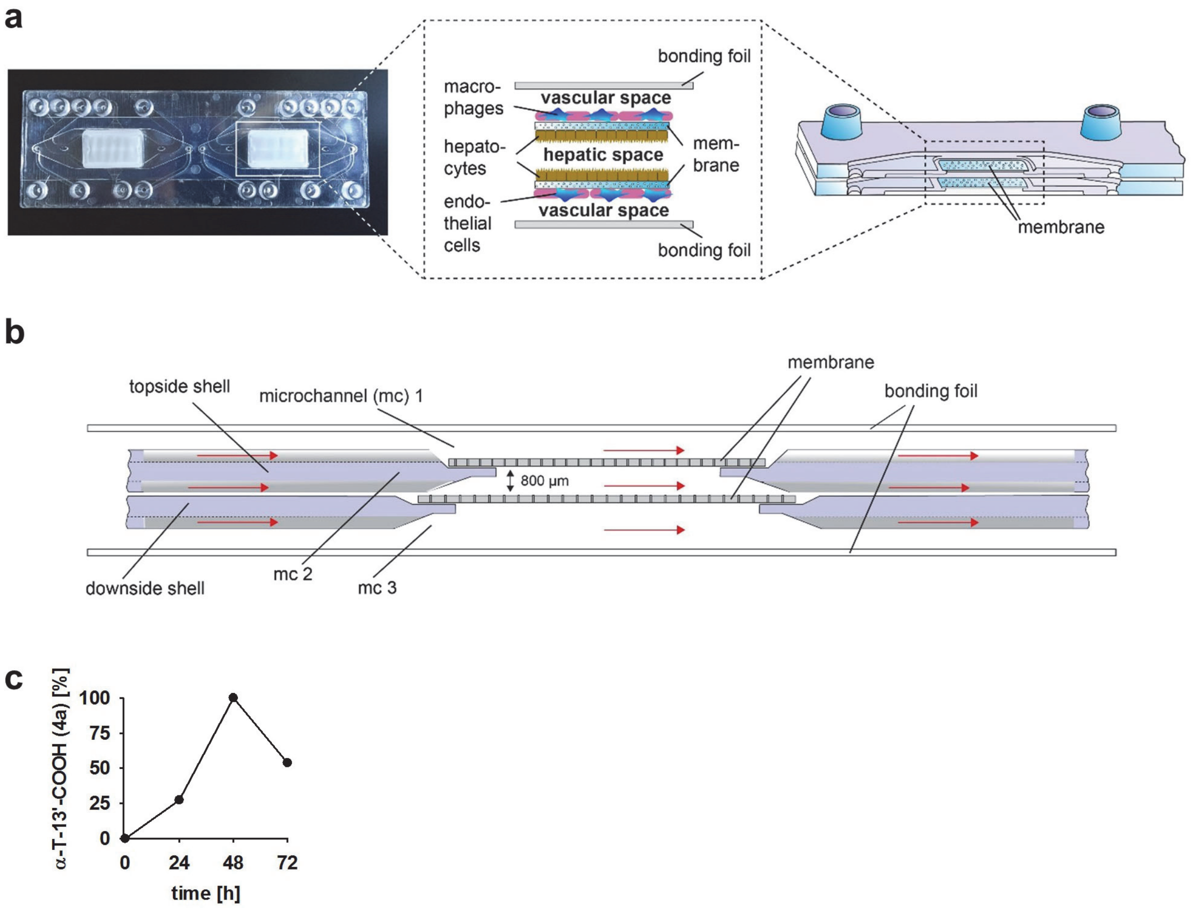

Supplementary Fig. 4 Design of the human liver-on-chip and α-T-13'-COOH (**4a**) biosynthesis. **a** Composition of the biochip-based human liver model. **b** Cross section of the microfluidic biochip. Vascular layers comprised of HUVEC and differentiated primary human monocyte-derived macrophages were cultured statically in micro-channels 1 and 3. Micro-channel 2 was used to build up a hepatic compartment comprised of human HepaRG hepatocytes and human LX-2 stellate cells. **c** Time-dependent production of α-T-13'-COOH (**4a**) by differentiated HepaRG cells (HepaRG biochip). Mean from n = 2 independent experiments.

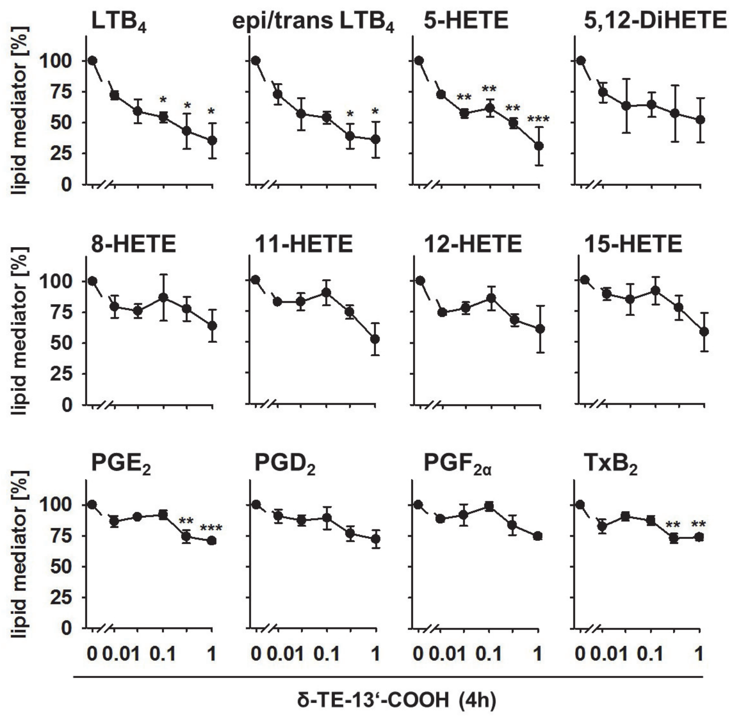

Supplementary Fig. 5 δ-TE-13'-COOH (**4h**) preferentially inhibits the formation of 5-LO-derived products within eicosanoid biosynthesis. Effect of δ-TE-13'-COOH (**4h**) on the eicosanoid profile in A23187/AA-treated monocytes that were pre-activated with LPS. (Di)HETE, (di)hydroxy-eicosatetraenoic acid; Tx, thromboxane. Mean ± s.e.m.; n = 3 independent experiments. *$P < 0.05$, **$P < 0.01$, ***$P < 0.001$ vs. vehicle control; repeated measures one-way ANOVA + Tukey HSD *post hoc* tests.

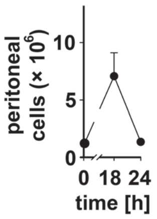

Supplementary Fig. 6 Time-dependent immune cell infiltration into the peritoneal cavity in zymosan-induced murine peritonitis. Mean + s.e.m. for n = 10 (0 h), n = 4 (18 h), n = 6 (24 h) mice.

0 100 200
[% w/o]

Lipid mediator	Group	exudate: w/o	exudate: a-T-13'-COOH (**4a**)	exudate: zileuton	plasma: w/o	plasma: a-T-13'-COOH (**4a**)	plasma: zileuton
PGE_2	COX	68	92	84	42	36	32
PGD_2	COX	19	27	24	60	25	16
$PGF_{2α}$	COX	12	18	18	n.d.	n.d.	n.d.
TxB_2	COX	108	142	125	102	106	70
12-HHT	COX	9	11	11	37	8	6
11-HETE	COX	48	72	63	201	132	131
11-HEPE	COX	4	4	4	78	56	81
LTB_4	5-LO	2	8	5	n.d.	n.d.	n.d.
LTB_4 isomers	5-LO	8	14	10	n.d.	n.d.	n.d.
5-HETE	5-LO	43	64	53	434	436	478
5-HEPE	5-LO	9	9	8	322	292	464
5,15-DiHETE	5-LO / 12/15-LO	7	6	6	60	47	37
12-HETE	12/15-LO	368	532	485	4061	3362	3725
15-HETE	12/15-LO	120	156	154	488	373	411
12-HEPE	12/15-LO	28	32	36	815	476	777
15-HEPE	12/15-LO	32	42	46	126	86	126
7-HDoHE	12/15-LO	8	9	8	161	99	134
14-HDoHE	12/15-LO	237	287	258	3155	1855	2459
17-HDoHE	12/15-LO	47	62	61	374	238	293
PD1	12/15-LO	2	4	3	n.d.	n.d.	n.d.
PDX	12/15-LO	3	3	3	n.d.	n.d.	n.d.
AT-PD1	12/15-LO	2	4	3	n.d.	n.d.	n.d.
RvE3	12/15-LO	10	11	5	38	206	35
MaR2	12/15-LO	2	2	2	3	3	3
9-HODE	others	1392	1149	952	5307	4556	5629
13-HODE	others	4202	3886	3231	12588	9807	12539
8-HETE	others	23	25	26	229	184	173
18-HEPE	others	9	10	11	215	212	331
4-HDoHE	others	12	9	10	262	219	278
13-HDoHE	others	17	18	16	285	203	248
21-HDoHE	others	26	22	18	306	318	260
AA	free PUFAs	37438	41788	41018	523754	509060	499960
EPA	free PUFAs	4592	4210	4716	182595	163214	191317
DHA	free PUFAs	23513	24582	24079	490699	433429	429026

Supplementary Fig. 7 Effect of α-T-13'-COOH (**4a**) on the lipid mediator profiles of peritoneal exudates and plasma during resolution of mouse peritonitis. Vehicle (DMSO), α-T-13'-COOH (**4a**) or zileuton (10 mg/kg, i.p., each) were administered to mice, which were killed at 18 h post zymosan injection as indicated in **Fig. 5**. Lipid mediator profiles were analyzed in the exudate and plasma by UPLC-MS/MS. Mean from n = 6 mice. The color scale refers to changes in the percentage of lipid mediators vs. vehicle control. The inserted values give the concentrations of lipid mediators in pg/ml. Tx, thromboxane; HTT, hydroxy-heptadecatrienoic acid; HEPE, hydroxy-eicosapentaenoic acid; (Di)HETE, (di)hydroxy-eicosatetraenoic acid; HDoHE, hydroxy-docosahexaenoic acid; (AT)-P, (apirin-triggerd) protectin; Rv, resolvin; MaR, maresin; HODE, hydroxy-octadecadienoic acid; EPA, eicosapentaenoic acid; DHA, docosahexaenoic acid.

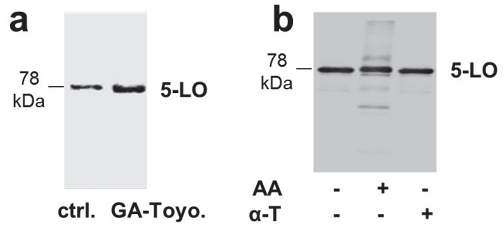

Supplementary Fig. 8 Uncropped Western blots. **a**, **b** Uncropped Western blots from **Fig. 1e**. (**a**) and **Fig. 1f** (**b**).

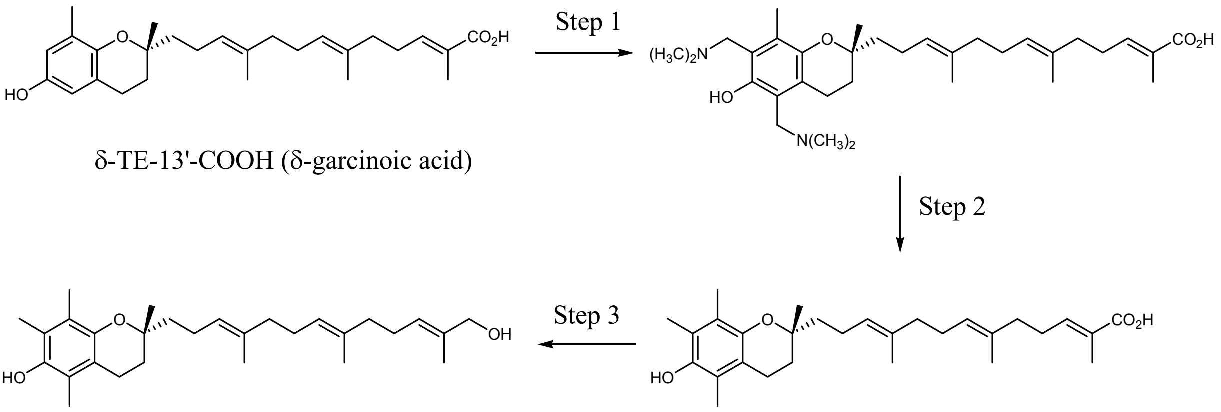

α-TE-13'-CH_2OH (α-deoxyamplexichromanol) α-TE-13'-COOH (α-garcinoic acid)

Supplementary Fig. 9. Multi-step strategy for the semi-synthesis of α-TE-13’-CH_2OH (**2e**)

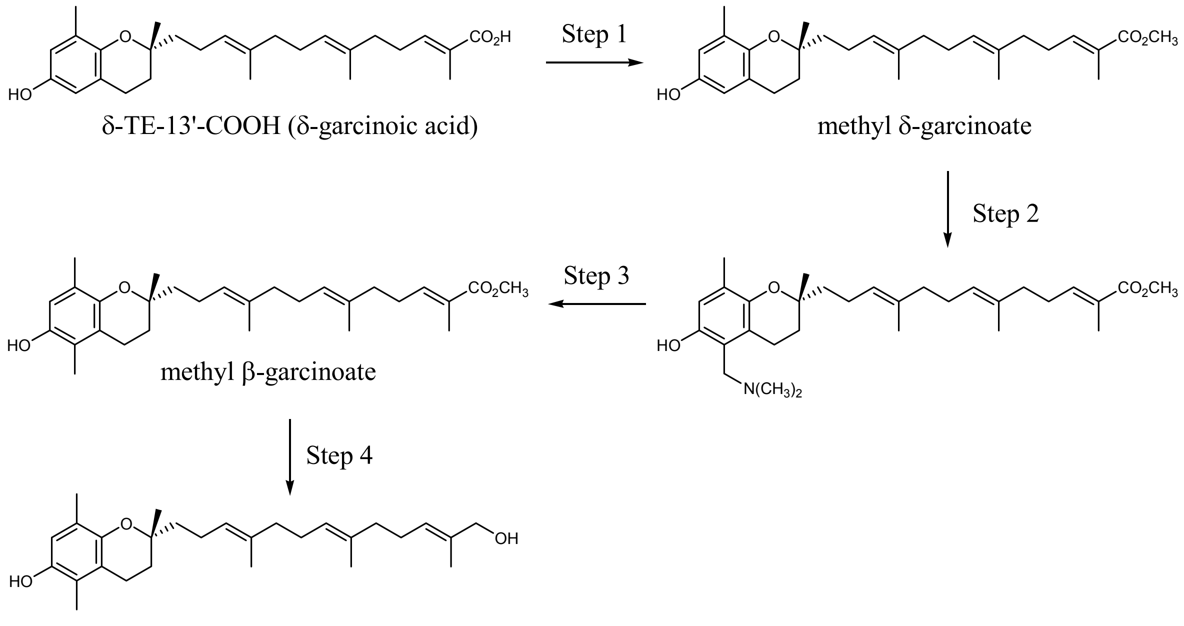

β-TE-13'-CH_2OH (β-deoxyamplexichromanol)

Supplementary Fig. 10. Multi-step strategy for the semi-synthesis of β-TE-13'-CH_2OH (**2f**)

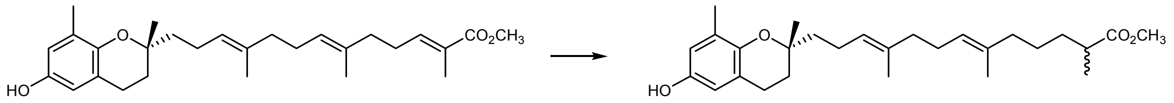

Supplementary Fig. 11. Semi-synthesis of methyl 19,20-dihydro-δ-garcinoate

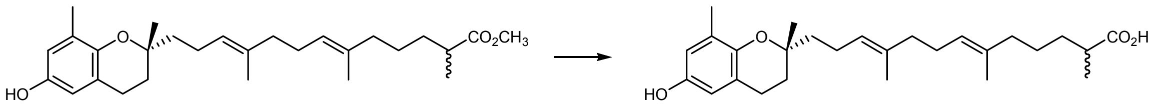

Supplementary Fig. 12. Semi-synthesis of δ-DE-13'-COOH (**4l**).

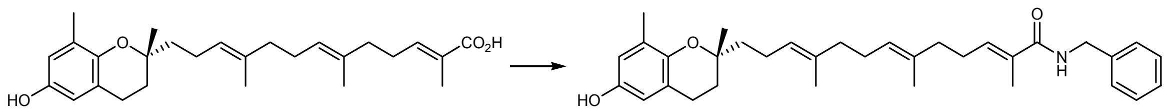

Supplementary Fig. 13. Semi-synthesis of δ-TE-13’-CONHBz (**7**).

Supplementary Tables

Supplementary Table 1 Docking scores for Ts, TEs, LCMs and vitamin E derivatives

Ts, TEs and LCMs

	T				TE			
	α (**a**)	β (**b**)	γ (**c**)	δ (**d**)	α (**e**)	β (**f**)	γ (**g**)	δ (**h**)
vitamin E (**1**)	56.6	59.7	59.0	55.8	58.9	48.9	56.2	59.8
13‘-CH_2OH (**2**)	60.2			52.3	50.1	51.6		64.7
12’a-CH_2OH (**3**)							51.2	68.7
13‘-COOH (**4**)	50.0			52.1	45.8	52.5	47.8	51.1

Derivatives

α-T-5‘-COOH (**5**)	57.1
α-T-3‘-COOH (**6**)	61.3
δ-TE-13‘-CONHBz (**7**)	46.0
δ-TE-12a‘/13‘-diCH_2OH (**8**)	62.2
γ-TE-12a‘/13‘-diCH_2OH (**9**)	55.9
adduct (**10**)	-0,8

Ligands were docked into the proposed LCM binding site at the interface of the regulatory C2-like domain and catalytic domain of 5-LO, and the highest score for each ligand is given. The ChemPLP scoring function is designed to predict ligand binding and includes a term for the binding free energy as well as other binding parameters[1].

Supplementary Table 2 Amino acid sequence alignment of human LO isoenzymes

```
                   13                   102
5-LO      YTVTVATGSQWFAGTDDY   -GDYIEFPCYRWITGDVEVVLRDGRAKLARDD
12-LO-1   YRIRVATGAWLFSGSYNR   ACAEVAFPCYRWVQGEDILSLPEGTARLPGDN
12R-LO    YKVRVATGTDLLSGTRDS   NGRIYHFPAYQWMDGYETLALREATGKTTADD
15-LO-1   YRIRVSTGASLYAGSNNQ   AGDEVRFPCYRWVEGNGVLSLPEGTGRTVGED
15-LO-2   FRVRVSTGEAFGAGTWDK   RGGHLLFPCYQWLEGAGTLVLQEGTAKVSWAD
          : : *:**    :*: :          **.*:*: *   : * :. .:    :
```

Supplementary Table 3 Conditions for the quantitation of LCMs by UPLC-MS/MS

LCM	transition	collision energy [eV]	external standard	lower limit of quantitation [nM][a]
α-T-3’-COOH (**6a**)	277 → 233	-25	α-T-3’-COOH (**6a**)	2
α-T-5‘-COOH (**5a**)	319 → 150	-24	α-T-13‘-COOH (**4a**)	/
α-T-7‘-COOH (**13a**)	347 → 163	-24	α-T-13‘-COOH (**4a**)	/
α-T-9‘-COOH (1**2a**)	389 → 163	-46	α-T-13‘-COOH (**4a**)	/
α-T-11‘-COOH (**11a**)	417 → 163	-46	α-T-13‘-COOH (**4a**)	/
α-T-13‘-COOH (**4a**)	459 → 163	-46	α-T-13‘-COOH (**4a**)	1
	459 → 150	-43		
	459 → 295	-40		
α-T-13‘-CH_2OH (**2a**)	445 → 163	-40	α-T-13‘-CH_2OH (**2a**)	250
γ-T-3’-COOH (**6c**)	263 → 219	-25	γ-T-3’-COOH (**6c**)	1
γ-T-13‘-COOH (**4c**)	445 → 149	-46	α-T-13‘-COOH (**4a**)	/
γ-T-13‘-CH_2OH (**2c**)	431 → 149	-40	α-T-13‘-CH_2OH (**2a**)	/
γ-TE-13‘-COOH (**4g**)	439 → 149	-40	γ-TE-13‘-COOH (**4g**)	0.5
γ-DE-13‘-COOH (**4k**)	441 → 149	-40	γ-TE-13‘-COOH (**4g**)	/
γ-TE-13‘-CH_2OH (**2g**)	425 → 149	-30	γ-TE-12a‘-CH_2OH (**3g**)	4
δ-T-3’-COOH (**6d**)	249 → 205	-25	δ-T-3’-COOH (**6d**)	2
δ-T-13‘-COOH (**4d**)	431 → 135	-46	δ-T-13‘-COOH (**4d**)	4
δ-TE-13‘-COOH (**4h**)	425 → 135	-40	δ-TE-13‘-COOH (**4h**)	1
δ-DE-13‘-COOH(**4l**)	427 → 135	-40	δ-TE-13‘-COOH (**4h**)	/
δ-T-13‘-CH_2OH (**2d**)	417 → 135	-46	δ-T-13‘-CH_2OH (**2d**)	60
δ-TE-13‘-CH_2OH (**2h**)	411 → 135	-30	δ-TE-13‘-CH_2OH (**2h**)	16

[a] signal-to-noise ratio ≥ 3

Supplementary Table 4 Plasma and exudate levels of vitamin E metabolites in mouse peritonitis

treatment (p.o.)	dose [mg/kg]	α-T (**1a**) metabolites [nM]							
		13'-COOH		11'-COOH		9'-COOH		7'-COOH	
		plasma	exudate	plasma	exudate	plasma	exudate	plasma	exudate
w/o	/	17.4±3.7	146.3±18.8	0.3±0.1	1.1±0.1	0.2±0.1	0.3±0.1	n.d.	n.d.
α-T (**1a**)	50	20.8±3.3	233.1±21.5 *	0.9±0.3	4.0±0.5 **	0.5±0.2	0.7±0.2 *	0.0±0.0	0.1±0.0
α-T (**1a**)/ sesamin	50/250	12.0±1.6	210.9±33.9	0.0±0.0	0.0±0.0	0.0±0.0	0.0±0.0	n.d.	n.d.
sesamin	250	15.6±3.1	160.1±39.7	0.4±0.0	1.4±0.3	0.6±0.2	7.6±1.7	n.d.	n.d.
zileuton	30	7.1±1.2	108.8±27.4	0.4±0.1	1.7±0.3	1.0±0.1	14.1±3.6 *	n.d.	n.d .

Concentrations of α-T metabolites (nM) are given as mean ± s.e.m.; n = 6 mice/group. **P* < 0.05, ***P* < 0.01 vs. vehicle control; one-way ANOVA + Tukey HSD *post hoc* tests.

Supplementary Table 5 Sequence of wildtype 5-LO and its site-directed mutants in the expression vectors 5-LO_wt, 5-LO_3W and 5-LO_Arg101Asp

```
1                                          W13                    60
ATGCCCTCCTACACGGTCACCGTGGCCACTGGCAGCCAGTGGTTCGCCGGCACTGACGAC     wt
ATGCCCTCCTACACGGTCACCGTGGCCACTGGCAGCCAGTGGTTCGCCGGCACTGACGAC     Arg101Asp
ATGCCCTCCTACACGGTCACCGTGGCCACTGGCAGCCAGGCGTTCGCCGGCACTGACGAC     3W

181                                              W75              240
GAACTGGGCGAGATCCAGCTGGTCAGAATCGAGAAGCGCAAGTACTGGCTGAATGACGAC     wt
GAACTGGGCGAGATCCAGCTGGTCAGAATCGAGAAGCGCAAGTACTGGCTGAATGACGAC     Arg101Asp
GAACTGGGCGAGATCCAGCTGGTCAGAATCGAGAAGCGCAAGTACGCGCTGAATGACGAC     3W

241                                                               300
TGGTACCTGAAGTACATCACGCTGAAGACGCCCCACGGGGACTACATCGAGTTCCCCTGC     wt
TGGTACCTGAAGTACATCACGCTGAAGACGCCCCACGGGGACTACATCGAGTTCCCCTGC     Arg101Asp
TGGTACCTGAAGTACATCACGCTGAAGACGCCCCACGGGGACTACATCGAGTTCCCCTGC     3W

301 R101 W102
TACCGC TGGATCACCGGCGAT                                           wt
TACGAC TGGATCACCGGCGAT                                           Arg101Asp
TACCGC GCGATCACCGGCGAT                                           3W
```

Supplementary Table 6 List of primer sequences

name	sequence
reverse primer for 5-LO sequencing	5’-GTTTTTGCCGTGTTTCCAGT-3’

Supplementary Notes

Supplementary Note 1

General Experimental Procedures

All solvents were dried and distilled before use. Reactions were run under inert nitrogen atmosphere. Unless otherwise stated, materials obtained from commercial suppliers were used without further purification. ^{1}H-NMR and ^{13}C-NMR were recorded on a JEOL JNM-ECZS400 spectrometer in deuterated chloroform and calibrated using the solvent resonance as internal reference. Chemical shifts δ are given in ppm, and coupling constants J are given in Hz. Complete ^{1}H NMR assignments have been determined through 1D and 2D (HMQC, HMBC, NOESY) NMR experiments. IR spectra were recorded on a Thermo Scientific Nicolet iS5 FT-IR Spectrometer and are reported in terms of frequency of absorption (cm^{-1}). Mass spectrometric analyses were performed on a JMS-700 (JEOL LTD) double focusing mass spectrometer with reversed geometry, equipped with a FAB source. Column chromatography was performed using silica gel 60A (particle size 40-63 µm) purchased from Fisher Scientific. Flash chromatography purifications using pre-packed columns (silica or octadecylsilyl silica, 4 g to 330 g) were realized on a CombiFlash Rf-200 equipped with a gradient pump, a column station with a DASi introduction system, a multi-wavelength UV detector, a fraction collector and a software to control the device. HPLC-DAD analyses were performed with a Waters Alliance HPLC system (Milford, CT, USA) equipped with a quaternary HPLC pump, degasser, autosampler, and PDA detector (Milford, CT, USA). HPLC-ELSD analyses were performed with a LC-2030 3D Prominence-i system (Shimadzu, Kyoto, Japan) equipped with a quaternary low-pressure gradient solvent delivery LC-2030 pump with a high-pressure switching valves, a LC-2030 vacuum degasser, both a ELSD-LT90 detector and a high sensitivity LC- 2030 PDA detector, a LC-2030 auto sampler and a LC-2030 column oven. Reactions under microwave irradiation were performed in the Monowave 300 (Anton Paar), equipped with the MAS 24 Autosampler, using borosilicate glass vials with snap caps. Purity of the original semisynthetic compounds described in this section has been determined using HPLC-ELSD analyses.

Semi-synthesis of α-TE-13'-CH_2OH (2e)

The multi-step strategy for the semi-synthesis of α-TE-13'-CH_2OH (**2e**) is shown in **Supplementary Fig. 9**.

Step 1:

5,7-bis(dimethylaminomethyl)-δ-garcinoic acid: *N*,*N*,*N'*,*N'*-tetramethyldiaminomethane TMDA (2.6 ml, 18.8 mmol, 20 equiv.) and paraformaldehyde (563 mg, 18.8 mmol, 20 equiv.) were added to a solution of δ-TE-13'-COOH (δ-garcinoic acid, **4h**; 1.4 g, 0.94 mmol, 1 equiv.) in 1,4-dioxane (12 ml). The reaction mixture was heated at 140°C under microwave irradiation for 40 min before quenching the reaction with water. The resulting mixture was extracted three times with dichloromethane (DCM) and the combined organic layers were washed with brine, dried over anhydrous Na_2SO_4, filtered and concentrated under reduced pressure. The brown oily crude product is used without further purification.

^1H NMR (400 MHz, $CDCl_3$): 6.35 (t, J = 7.2 Hz, 1H), 5.07 (t, J = 7.2 Hz, 1H), 5.05 (t, J = 7.2 Hz, 1H), 3.96 (s, 2H), 3.86 (s, 2H), 2.61 (m, 2H), 2.54 (s, 6H), 2.48 (s, 6H), 2.25-1.91 (m, 10H), 2.16 (s, 3H), 2.10 (s, 3H), 1.82-1.63 (m, 2H), 1.70 (s, 3H), 1.56-1.42 (m, 2H), 1.52 (s, 3H), 1.49 (s, 3H), 1.22 (s, 3H); ^{13}C NMR (100 MHz, $CDCl_3$): 177.7, 151.7, 145.8, 137.2, 133.8, 136.3, 134.3, 125.8, 125.6, 124.6, 122.0, 118.4, 116.2, 75.8, 56.9, 56.5, 44.0, 43.9, 40.7, 40.2, 39.9, 32.4, 28.4, 27.4, 24.3, 23.3, 21.2, 16.3, 16.0, 13.9, 12.1; HRMS (FAB): (m/z) calcd. for $C_{33}H_{52}N_2O_4$ $[M + H]^+$ 541.4000, found 541.3997.

Step 2:

α-TE-13'-COOH (α-garcinoic acid, **4e**): $NaBH_3CN$ (1.1 g, 17.2 mmol, 20 equiv.) was added to a solution of 5,7-bis(dimethylaminomethyl)-δ-garcinoic acid (466 mg, 0.86 mmol, 1 equiv.) in ethanol (14 ml). The reaction mixture was heated at 145°C under microwave irradiation for 30 min. Then the reaction was quenched with a 10% aqueous HCl solution (10 ml). The resulting mixture was extracted three times with diethyl ether (Et_2O; 20 ml) and the combined organic layers were successively washed with water, brine, dried over anhydrous Na_2SO_4, filtered and concentrated under reduced pressure. The residue was purified via column chromatography on silica gel eluted with a petroleum ether (PE)/acetone/DCM mixture (8:1:1) as a mobile phase to afford α-TE-13'-COOH (α-garcinoic acid, **4e**) (343 mg, 0.75 mmol, 80% over two steps from δ-garcinoic acid, δ-TE-13'-COOH, **4h**) as a pale oil.

^1H NMR (δ ppm): 6.88 (t, J = 6.8 Hz, 1H), 5.13 (t, J = 3.8 Hz, 2H), 2.61 (t, J = 6.8 Hz, 2H), 2.31-2.25 (m, 2H), 2.16 (s, 3H), 2.15-2.04 (m, 6H), 2.12 (s, 3H), 2.11 (s, 3H), 1.99-1.96 (m,

2H), 1.85-1.75 (m, 2H), 1.82 (s, 3H), 1.67-1.42 (m, 2H), 1.60 (s, 6H), 1.25 (s, 3H); ^{13}C NMR (δ ppm): 173.2, 146.6, 145.1, 144.7, 136.0, 133.8, 127.0, 125.3, 124.7, 122.8, 121.2, 118.6, 117.4, 74.4, 39.7, 39.6, 38.2, 31.7, 27.7, 26.7, 23.8, 22.3, 20.9, 16.1, 16.0, 12.4, 12.1, 11.9, 11.4; HRMS (FAB): (m/z) calcd. for $C_{29}H_{42}O_4$ $[M]^+$ 454.3078, found 454.3084.

Step 3:

α-TE-13'-CH_2OH (α-deoxyamplexichromanol, **2e**): A solution of α-TE-13'-COOH (α-garcinoic acid, **4e**; 71 mg, 0.38 mmol, 1 equiv.) in tetrahydrofuran (9 ml) was added to a solution of $LiAlH_4$ (29 mg, 0.76 mmol, 2 equiv.) in tetrahydrofuran (9 ml) at 0°C. The reaction mixture was stirred at room temperature for 2 h. Then it was quenched with two drops of a solution of NaOH (1M) prior to H_2O addition (5 ml). The resulting mixture was stirred for 15 min, and extracted three times with Et_2O (10 ml). The combined organic layers were washed with a saturated aqueous solution of Rochelle's salt (20 ml), dried over anhydrous Na_2SO_4, filtered and concentrated under reduced pressure. The residue was purified via column chromatography on silica gel eluted with a PE/acetone mixture (9:1) to afford α-TE-13'-CH_2OH (α-deoxyamplexichromanol, **2e**) (65 mg, 0.15 mmol, 39% yield) as an orange oil. Purity (HPLC-ELSD): 99%. TLC (petroleum ether:acetone, 80:20 v/v): $R_f = 0.39$; ^{1}H NMR (400 MHz, $CDCl_3$): 5.38 (ddq, J = 1.3 Hz, 5.6 Hz, 8.3 Hz, 1H), 5.12 (m, 2H), 3.99 (s, 2H), 2.61 (t, J = 6.9 Hz, 2H), 2.18-2.04 (m, 6H), 2.16 (s, 3H), 2.12 (s, 3H), 2.11 (s, 3H), 2.08 (s, 3H), 2.01-1.95 (m, 4H), 1.85-1.75 (m, 2H), 1.66 (s, 3H), 1.65-1.51 (m, 2H), 1.59 (s, 6H), 1.25 (s, 3H); ^{13}C NMR (100 MHz, $CDCl_3$): 145.6, 144.7, 135.1, 134.8, 134.7, 126.3, 124.6 (2C), 122.8, 121.2, 118.6, 117.4, 74.4, 69.2, 39.8, 39.6, 39.4, 31.7, 26.7, 26.4, 23.9, 22.4, 20.9, 16.1, 16.0, 13.8, 12.4, 11.9, 11.1; IR (ATR): 3376, 2921, 1450, 1377, 1253, 1164, 1085, 856 cm^{-1}; HRMS (FAB): (m/z) calcd. for $C_{29}H_{44}O_3$ $[M]^+$ 440.3285, found 440.3295.

Semi-synthesis of β-TE-13'-CH_2OH (2f)

The multi-step strategy for the semi-synthesis of β-TE-13'-CH_2OH (**2f**) is shown in **Supplementary Fig. 10**.

Step 1:

Methyl-δ-garcinoate: $NaHCO_3$ (434 mg, 5.16 mmol, 4 equiv.) and iodomethane (321 µl, 5.16 mmol, 4 equiv.) were added to a solution of *δ*-garcinoic acid (550 mg, 1.29 mmol, 1 equiv.) in DMF (10 ml). The reaction mixture was heated at 120°C under microwave irradiation for 45

minutes. Then the reaction was quenched with water. The resulting mixture was extracted with Et_2O (three times 20 ml) and the combined organic layers were washed with water (150 ml) and brine (50 ml), dried over anhydrous Na_2SO_4, filtered and concentrated under reduced pressure. The residue was purified by silica gel column chromatography eluted with PE/acetone (9:1) to afford methyl δ-garcinoate (542 mg, 1.23 mmol, 95% yield) as a yellow oil.

^{1}H NMR (400 MHz, $CDCl_3$): 6.75 (td, J = 1.4 Hz , 7.3 Hz, 1H), 6.49 (d, J = 2.9 Hz, 1H), 6,39 (d, J = 2.9 Hz, 1H), 5.14-5.10 (m, 2H), 4.84 (s, OH), 3.74 (s, 3H), 2.69 (t, J = 6.9 Hz, 2H), 2.25 (dd, J = 7.5 Hz, 15.1 Hz, 2H), 2.12 (s, 3H), 2.10-2.04 (m, 6H), 1.98-1.95 (m, 2H), 1.83 (d, J = 1.3 Hz, 3H), 1.81-1.71 (m, 2H), 1.67-1.49 (m, 8H), 1.26 (s, 3H); ^{13}C NMR (100 MHz, $CDCl_3$): 169.0, 148.0, 146.0, 142.7, 135.0, 134.0, 127.4 (2), 125.1, 124.6, 121.3, 115.8, 112.7, 75.4, 51.9, 39.7, 39.6, 38.3, 31.5, 27.5, 26.6, 24.2, 22.6, 22.3, 16.2, 16.1, 16.0, 12.5; HRMS (FAB): (m/z) calcd for $C_{28}H_{40}O_4$ $[M]^+$ 440.2921, found 440.2924.

Step 2:

Methyl 5-dimethylaminomethyl-δ-garcinoate: TMDA (184 µl, 1.35 mmol, 3 equiv.) and paraformaldehyde (41 mg, 1.35 mmol, 3 equiv.) were added to a 6 ml ethanol solution of δ-methyl garcinoate (200 mg, 0.45 mmol, 1 equiv.). The reaction mixture was heated at 120°C under microwave irradiation for 60 min and then the reaction was quenched with water (10 ml). The resulting mixture was extracted three times with DCM (20 ml) and the combined organic layers were washed with brine (40 ml), dried over anhydrous Na_2SO_4, filtered and concentrated under reduced pressure. The brown oily crude product (199 mg) is used without further purification.

^{1}H NMR (400 MHz, $CDCl_3$): 6.74 (tq, J = 1.4 Hz, 7.3 Hz, 1H), 6.52 (s, 1H), 5.12 (t, J = 6.3 Hz, 2H), 3.72 (s, 3H), 3.57 (s, 2H), 2.59 (t, J = 6.9 Hz, 2H), 2.30 (s, 6H), 2.26-2.22 (m, 2H), 2.12 (s, 3H), 2.09-2.05 (m, 6H), 1.98-1.96 (m, 2H), 1.83 (s, 3H), 1.80-1.73 (m, 2H), 1.65-1.52 (m, 2H), 1.59 (s, 3H), 1.58 (s, 3H), 1.23 (s, 3H); ^{13}C NMR (100 MHz, $CDCl_3$): 168.9, 150.6, 144.7, 142.5, 135.2, 134.0, 127.5, 126.7, 125.2, 124.5, 118.5, 116.7, 116.4, 74.2, 57.8, 51.8, 44.6 (2C), 39.7, 39.6, 38.3, 31.6, 27.5, 26.7, 23.8, 22.3, 20.6, 16.3, 16.1, 16.0, 12.5; HRMS (FAB): (m/z) calcd for $C_{31}H_{47}NO_4$ $[M]^+$ 497.3500, found 497.3502.

Step 3:

Methyl β-garcinoate: $NaBH_3CN$ (75 mg, 1.2 mmol, 3 equiv.) was added to a solution of methyl 5-dimethylaminomethyl-δ-garcinoate (199 mg, 0.4 mmol, 1 equiv.) in ethanol (6 ml). The reaction mixture was heated at 120°C under microwave irradiation for 45 min. Then the

reaction was quenched with 10% aqueous HCl solution (5 ml). The resulting mixture was extracted three times with Et_2O (10 ml) and the combined organic layers were washed with water (30 ml), brine (30 ml), dried over anhydrous Na_2SO_4, filtered and concentrated under reduced pressure. The brown oily crude product is used without further purification.

^{1}H NMR (400 MHz, $CDCl_3$): 6.75 (tq, J = 1.3 Hz, 7.3 Hz, 1H), 6.49 (s, 1H), 5.13 (t, J = 7.4 Hz, 2H), 4.74 (br, OH), 3.74 (s, 3H), 2.61 (t, J = 6.8 Hz, 2H), 2.26 (dd, J = 7.5 Hz, 15.1 Hz, 2H), 2.15-2.05 (m, 6H), 2.11 (s, 3H), 2.09 (s, 3H), 1.99-1.95 (m, 2H), 1.87-1.76 (m, 2H), 1.84 (s, 3H), 1.67-1.51 (m, 2H), 1.60 (s, 6H), 1.26 (s, 3H); ^{13}C NMR (100 MHz, $CDCl_3$): 169.0, 146.0, 145.9, 142.6, 135.0, 133.9, 127.5, 125.2, 124.6, 124.0, 120.4, 119.4, 115.5, 74.3, 51.9, 39.7, 39.4, 38.3, 31.6, 27.5, 26.6, 23.9, 22.3, 20.9, 16.1, 16.0, 16.0, 12.5, 11.1; HRMS (FAB): (m/z) calcd for $C_{29}H_{42}O_4$ $[M]^+$ 454.3078, found 454.3080.

Step 4:

β-TE-13'-CH_2OH (β-deoxyamplexichromanol, **2f**): A solution of methyl β-garcinoate (182 mg, 0.40 mmol, 1 equiv.) in tetrahydrofuran (10 ml) was added to a solution of $LiAlH_4$ (31 mg, 0.80 mmol, 2 equiv.) in tetrahydrofuran (10 ml) at 0°C. After stirring 1.5 h at room temperature, the reaction was quenched with two drops of an aqueous solution of NaOH (1M) then H_2O (5 ml). The resulting mixture was stirred for 15 min, extracted three times with Et_2O (10 ml). The combined organic layers were washed with saturated aqueous solution of Rochelle's salt, dried over anhydrous Na_2SO_4, filtered and concentrated under reduced pressure. The residue was purified via column chromatography on silica gel eluted with a PE/acetone mixture (9:1) to afford β-TE-13'-CH_2OH (β-deoxyamplexichromanol, **2f**) (54 mg, 0.13 mmol, 29% yield from methyl δ-garcinoate) as a yellow oil.

Purity (HPLC-ELSD): 99%; TLC (petroleum ether:acetone, 80:20 v/v): R_f = 0.32; ^{1}H NMR (400 MHz, $CDCl_3$): 6.48 (s, 1H), 5.38 (ddq, J = 1.3 Hz, 5.6 Hz, 8.3 Hz, 1H), 5.11 (dd, J = 7.1 Hz, 16.1 Hz, 2H), 3.99 (s, 2H), 2.61 (t, J = 6.9 Hz, 2H), 2.14-2.05 (m, 6H), 2.11 (s, 3H), 2.08 (s, 3H), 1.98-1.95 (m, 4H), 1.87-1.74 (m, 2H), 1.66 (s, 3H), 1.63-1.50 (m, 2H), 1.59 (s, 6H), 1.25 (s, 3H); ^{13}C NMR (100 MHz, $CDCl_3$): 146.0, 145.9, 135.1, 134.8, 134.7, 126.4, 124.6, 124.3, 124.1, 120.4, 119.4, 115.4, 74.4, 69.2, 39.8, 39.4, 39.3, 31.6, 26.7, 26.4, 24.0, 22.3, 20.9, 16.7, 16.1, 16.0, 13.8, 11.1; IR (ATR): 3357, 2970, 2922, 1458, 1230, 1164, 1098, 1004 cm^{-1}; HRMS (FAB): (m/z) calcd for $C_{28}H_{42}O_3$ $[M]^+$ 426.3128, found 426.3131.

Semi-synthesis of δ-DE-13'-COOH (4l):

The semi-synthesis of methyl 19,20-dihydro-δ-garcinoate is shown in **Supplementary Fig. 11**, and the subsequent semi-synthesis of δ-DE-13'-COOH (**4l**) is shown in **Supplementary Fig. 12**.

Methyl 19,20-dihydro-δ-garcinoate: Magnesium shavings (156 mg, 6.4 mmol) were added to a solution of methyl *δ*-garcinoate (140 mg, 0.32 mmol) in distilled methanol (14 ml). The reaction mixture was stirred at room temperature for 24 h and then the reaction was quenched with water (5 ml). The resulting mixture was extracted with Et_2O (three times 10 ml) and the combined organic layers were washed with water (10 ml) and brine (10 ml), dried over anhydrous Na_2SO_4, filtered and concentrated under reduced pressure. The brown oily crude product was used for next step without further purification.
^{1}H NMR (400 MHz, $CDCl_3$): 6.48 (d, J = *3.0* Hz, 1H), 6.39 (d, J = *3.0* Hz, 1H), 5.10 (dt, J = 6.6 Hz, 10.3 Hz, 2H), 3.67 (s, 3H), 2.69 (t, J = 6.9 Hz, 2H), 2.44 (dd, J = 7.0 Hz, 13.0 Hz, 1H), 2.12 (s, 3H), 2.09-2.02 (m, 4H), 1.98-1.92 (m, 4H), 1.82-1.69 (m, 2H), 1.67-1.49 (m, 4H), 1.58 (s, 3H), 1.55 (s, 3H), 1.39-1.33 (m, 2H), 1.26 (s, 3H), 1.14 (d, J = 7.0 Hz, 3H); ^{13}C NMR (100 MHz, $CDCl_3$): 177.7, 148.0, 146.0, 135.2, 134.8, 127.4, 124.6, 124.4, 121.3, 115.8, 112.7, 75.4, 51.7, 39.8, 39.6, 39.5 (2C), 33.4, 31.5, 26.6, 25.6, 24.2, 22.6, 22.3, 17.2, 16.2, 16.0, 15.9; HRMS (FAB): (m/z) calcd for $C_{28}H_{42}O_4$ $[M]^+$ 442.3078, found 442.3078.

δ-DE-13'-COOH (**4l**)**:** 4 ml of a 2 M sodium hydroxide methanol solution were added to a solution of methyl 19,20-dihydro-δ-garcinoate (100 mg, 0.23 mmol) in methanol. The reaction mixture was heated at 70°C for 4 h and then the reaction was quenched with 10 ml of a 10% HCl aqueous solution. The resulting mixture was extracted with Et_2O (three times 10 ml) and the combined organic layers were washed with water (15 ml) and brine (15 ml), dried over anhydrous Na_2SO_4, filtered and concentrated under reduced pressure. The residue was purified by column chromatography eluted with PE/acetone (9:1) to afford δ-DE-13'-COOH (**4l**, 94 mg, 96%) as an orange oil.
Purity (HPLC-ELSD): 99%; TLC (petroleum ether:acetone:CH_2Cl_2, 70:20:10 v/v): R_f = 0.23; ^{1}H NMR (400 MHz, $CDCl_3$): 6.48 (d, J = 3 Hz, 1H), 6.38 (d, J = 3 Hz,1H), 5.14-5.07 (t, 2H), 2.67-2.71 (t, J = 6.8 Hz, 2H), 2.49-2.44 (q, 1H), 2.13 (s, 3H), 2.09-2.03 (m, 6H), 1.98-1.94 (m, 2H), 1.82-1.72 (m, 2H), 1.58 (s, 3H), 1.56 (s, 3H), 1.67-1.49 (m, 2H), 1.45-1.36 (m, 4H), 1.26 (s, 3H), 1.18-1.16 (d, J = 6.9 Hz, 3H); ^{13}C NMR (100 MHz, $CDCl_3$): 181.9, 147.8, 146.1, 135.2, 134.7, 127.5, 124.7, 124.5, 121.4, 115.8, 112.7, 75.5, 39.8, 39.7, 39.5, 39.2, 33.2, 31.5, 26.6,

25.5, 24.2, 22.6, 22.3, 17.0, 16.2, 16.0, 15.9; IR (ATR): 2927, 1703, 1609, 1465, 1378, 1219, 1097, 853 cm^{-1}; HRMS (FAB): (m/z) calcd for $C_{27}H_{40}O_4$ $[M]^+$ 428.2921, found 428.2930.

Semi-synthesis of δ-TE-13'-CONHBz (7)

The semi-synthesis of δ-TE-13'-CONHBz (**7**) is shown in **Supplementary Fig. 13**.

Benzylamine (57 µl, 0.53 mmol, 1.5 equiv.), diisopropylethylamine (183 µl, 1.05 mmol, 3 equiv.) and HBTU as a coupling agent (201 mg, 0.53 mmol, 1.5 equiv.) were added to a solution of δ-garcinoic acid (150 mg, 0.35 mmol, 1 eq) in 4 ml of dry DMF. The reaction mixture was stirred at room temperature for 18 h then the reaction was quenched with water (5 ml). The resulting mixture was extracted with Et_2O (three times 10 ml) and the combined organic layers were successively washed with water (15 ml) and brine (15 ml), dried over anhydrous Na_2SO_4, filtered and concentrated under reduced pressure. The crude residue was purified by column chromatography eluted with PE/acetone (9:1) to afford the benzylamide (160 mg, 0.31 mmol, 88% yield) as an oil.

Purity (HPLC-ELSD): 99%; TLC (petroleum ether:acetone:CH_2Cl_2, 70:20:10 v/v): R_f= 0.50; ^{1}H NMR (400 MHz, $CDCl_3$): 7.36-7.28 (m, 5H), 6.48 (d, J = 2.7 Hz, 1H), 6.38 (d, J = 2.7 Hz, 1H), 6.35 (t, J = 7.2 Hz 1H), 6.02 (br, 1H), 5.08 (dd, J = 6.2 Hz, 12.8 Hz, 2H), 4.51 (d, J = 5.6 Hz, 2H), 2.67 (t, J = 6.8 Hz, 2H), 2.19 (dd, J = 7.4 Hz, 15.0 Hz, 2H), 2.11 (s, 3H); 2.08-2.00 (m, 6H), 1.98-1.93 (m, 2H), 1.84 (s, 3H), 1.81-1.69 (m, 2H), 1.65-1.50 (m, 2H), 1.57 (s, 6H), 1.27 (s, 3H); ^{13}C NMR (100 MHz, $CDCl_3$): 169.7, 148.3, 145.8, 138.8, 138.4, 136.8, 134.2, 130.5, 128.9 (2C), 128.0 (2C), 127.7, 127.3, 125.0, 124.8, 121.2, 115.9, 112.8, 75.3, 44.0, 39.6, 39.1, 38.5, 31.5, 27.2, 26.4, 24.5, 22.6, 22.3, 16.2, 16.1, 15.9, 12.9; IR (ATR): 3328, 2924, 1659, 1617, 1528, 1469, 1219, 933 cm^{-1}; HRMS (FAB): (m/z) calcd for $C_{34}H_{45}NO_3$ $[M]^+$ 515.3394, found 515.3387.

^{1}H and ^{13}C NMR spectra

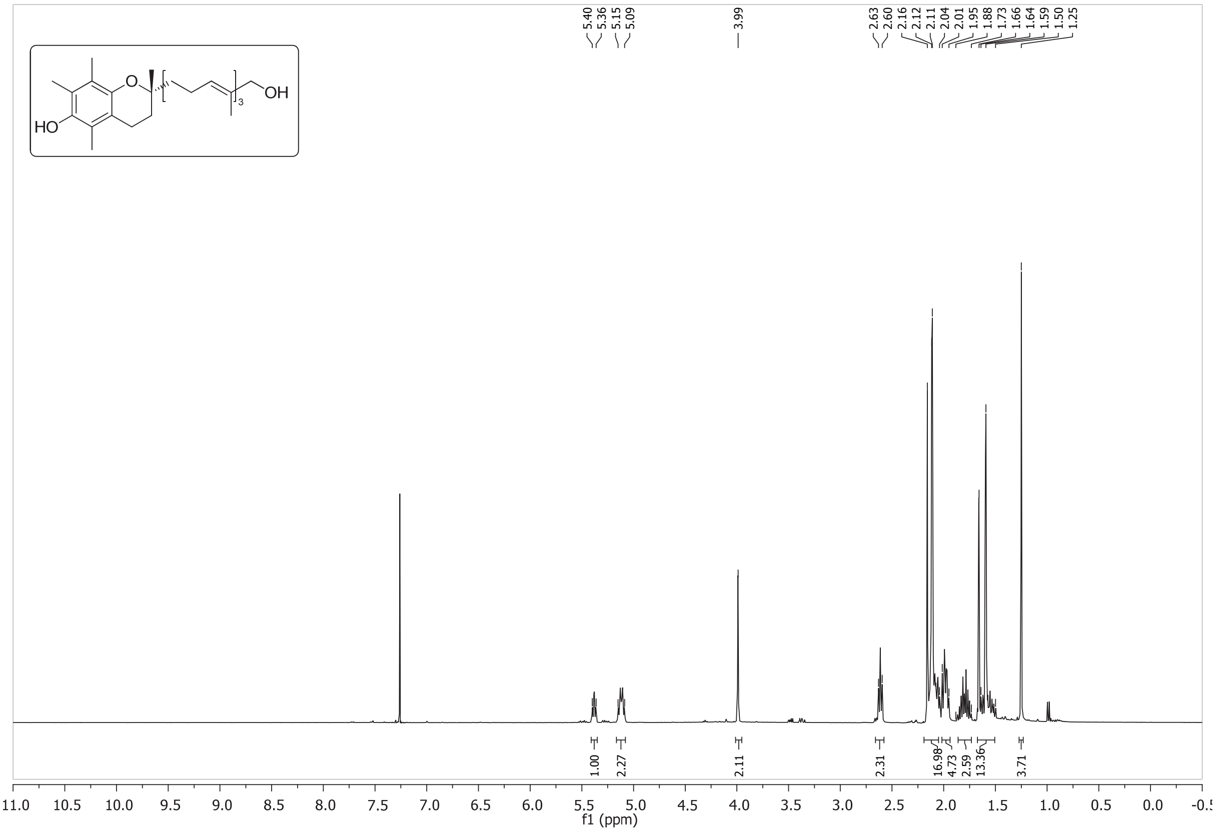

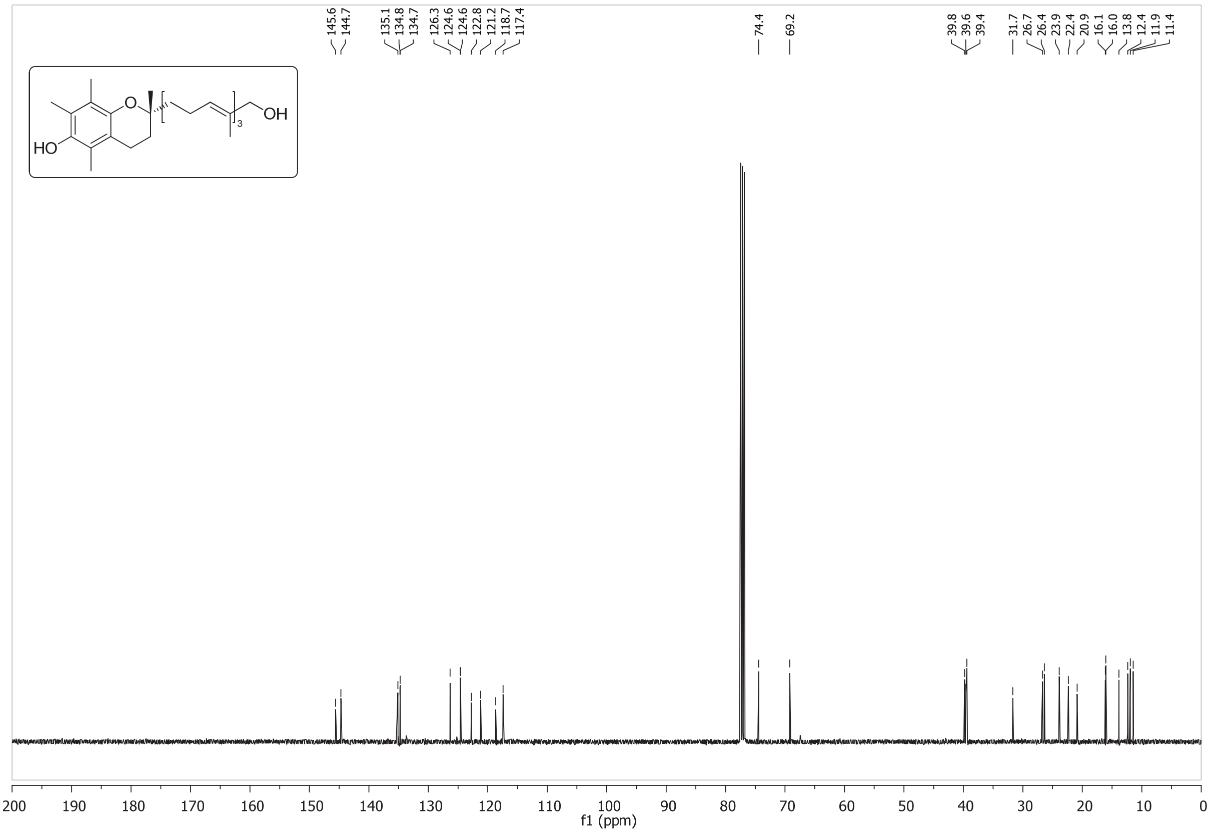

^{1}H and ^{13}C NMR spectra of α-TE-13’-CH_2OH **(2e)** in $CDCl_3$

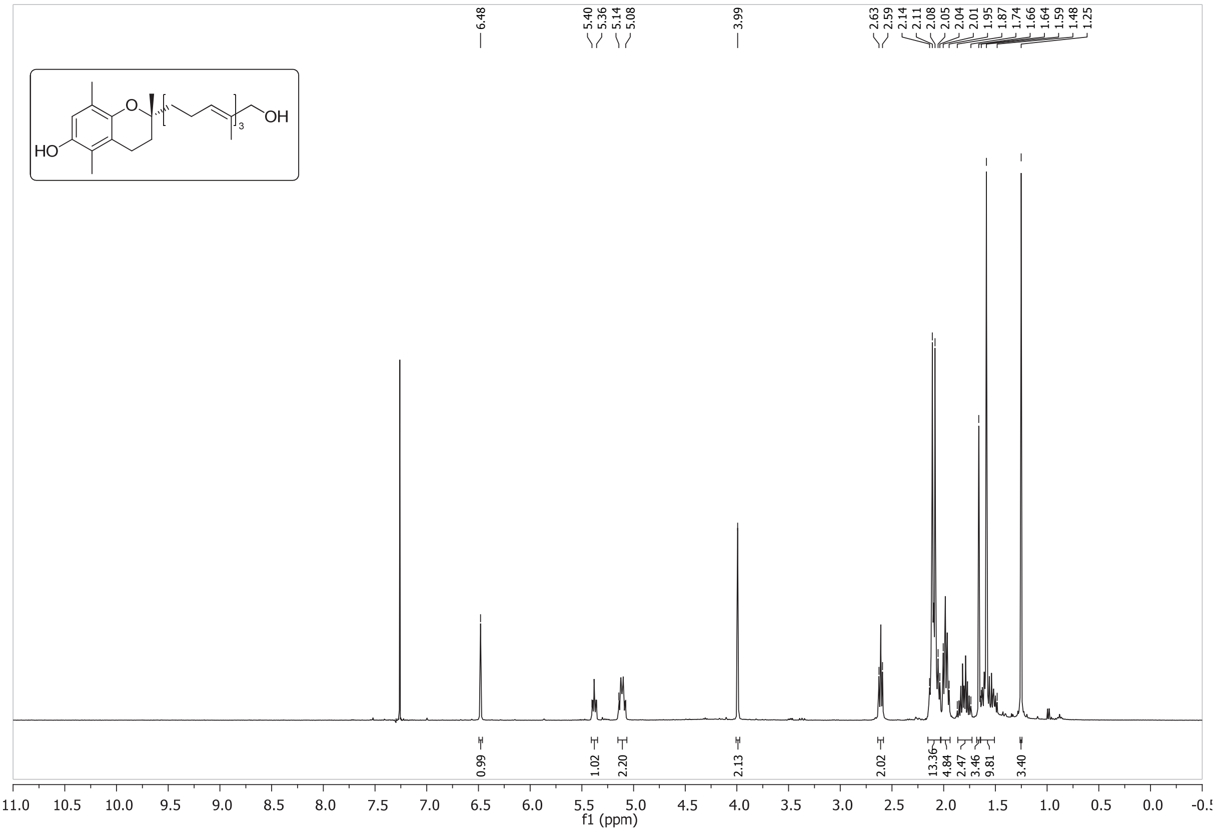

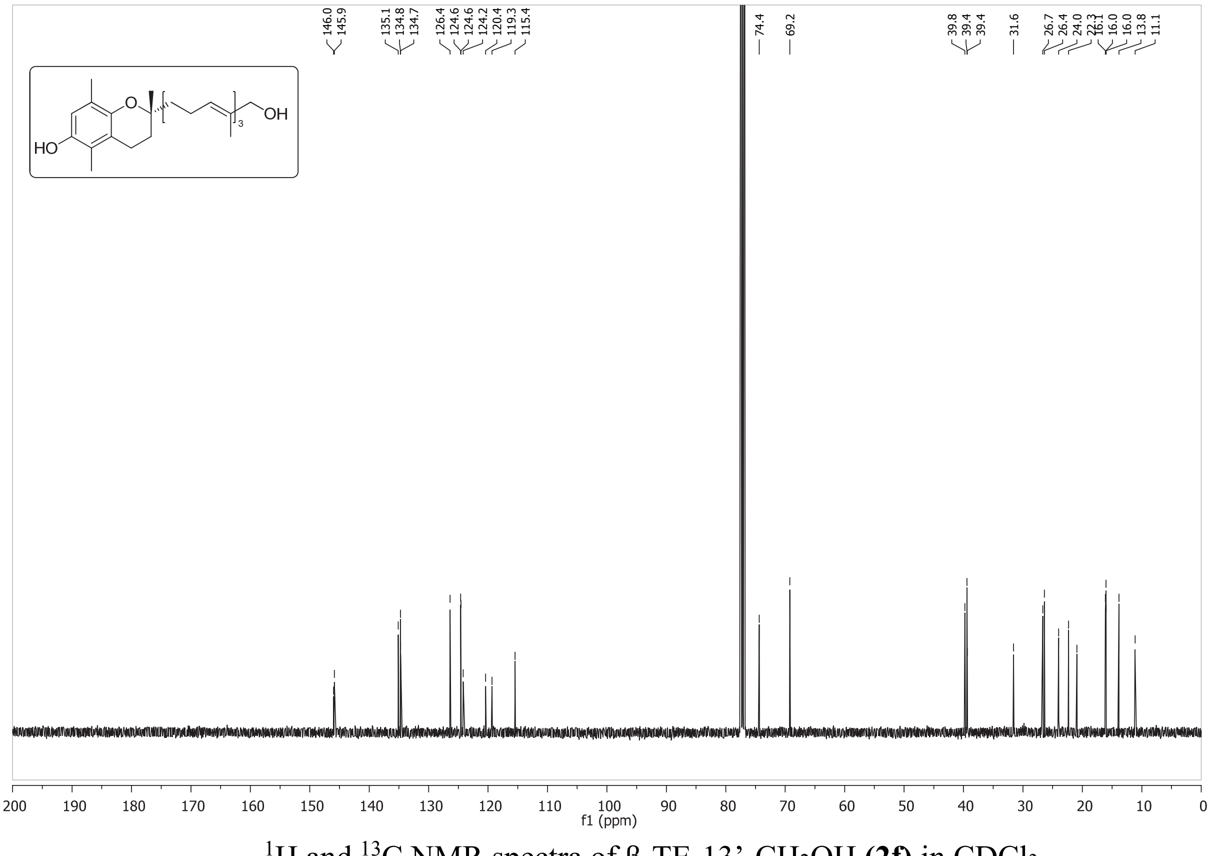

^{1}H and ^{13}C NMR spectra of β-TE-13'-CH_2OH **(2f)** in $CDCl_3$

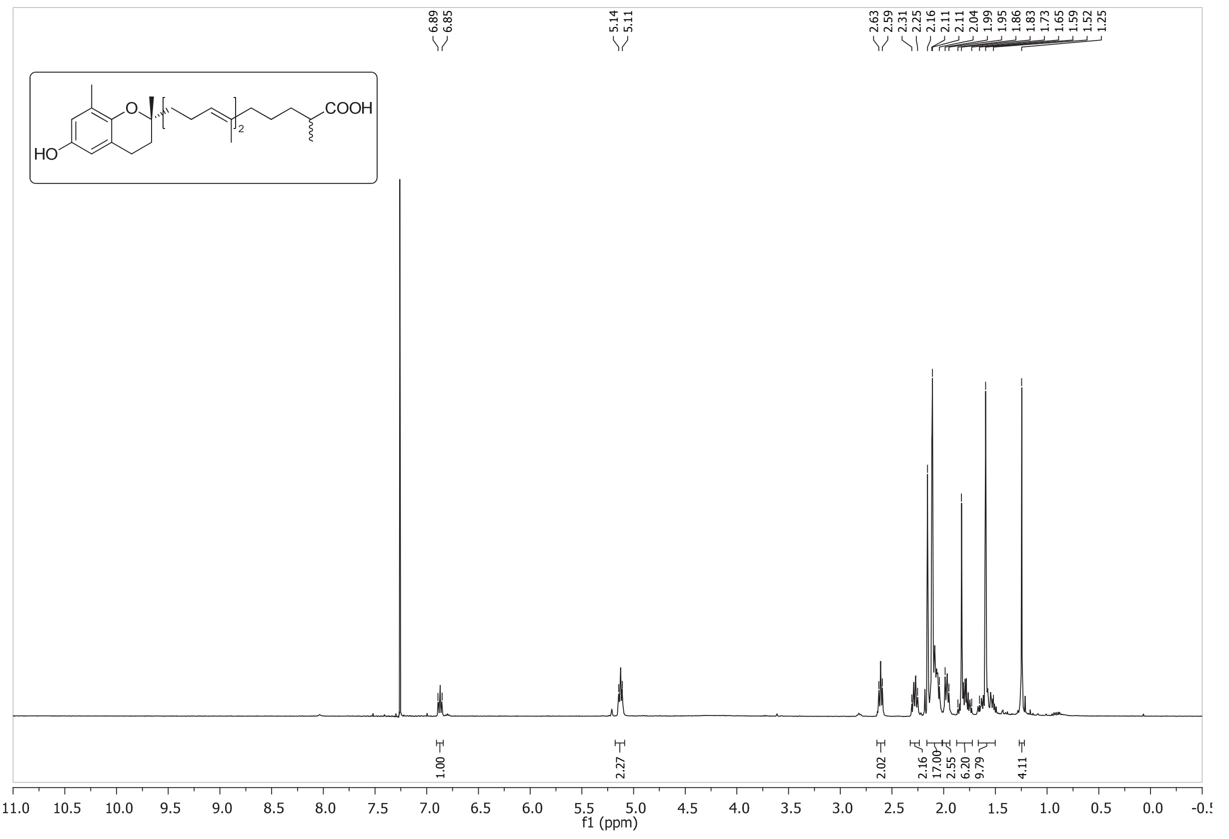

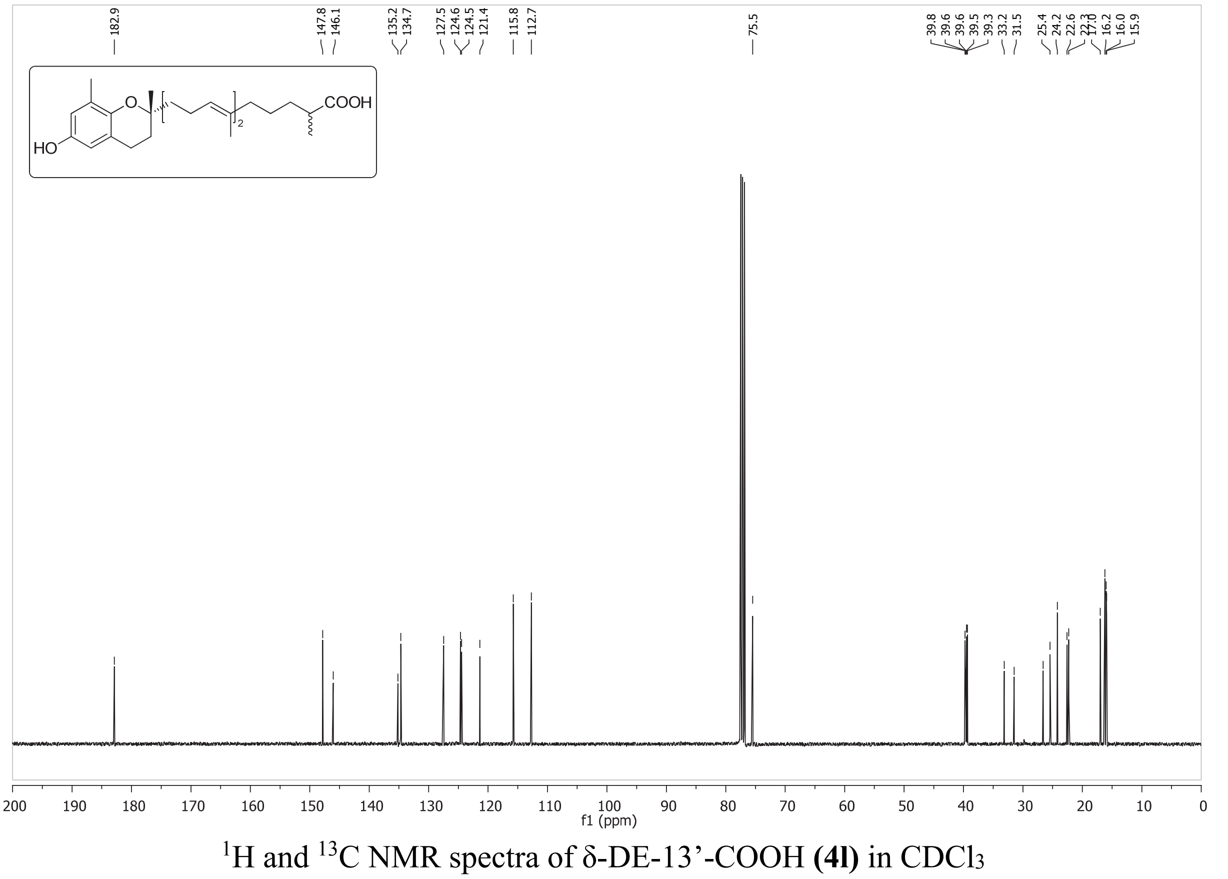

^{1}H and ^{13}C NMR spectra of δ-DE-13’-COOH **(4l)** in $CDCl_3$

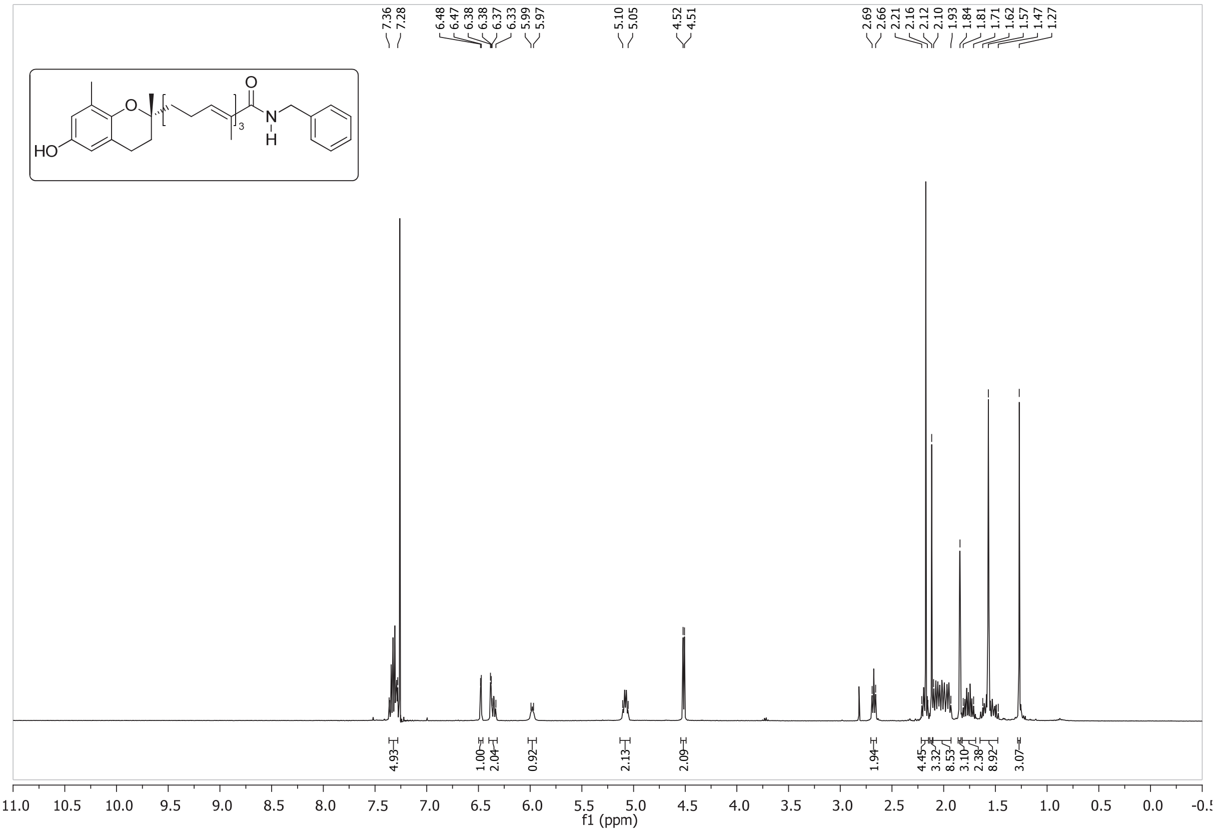

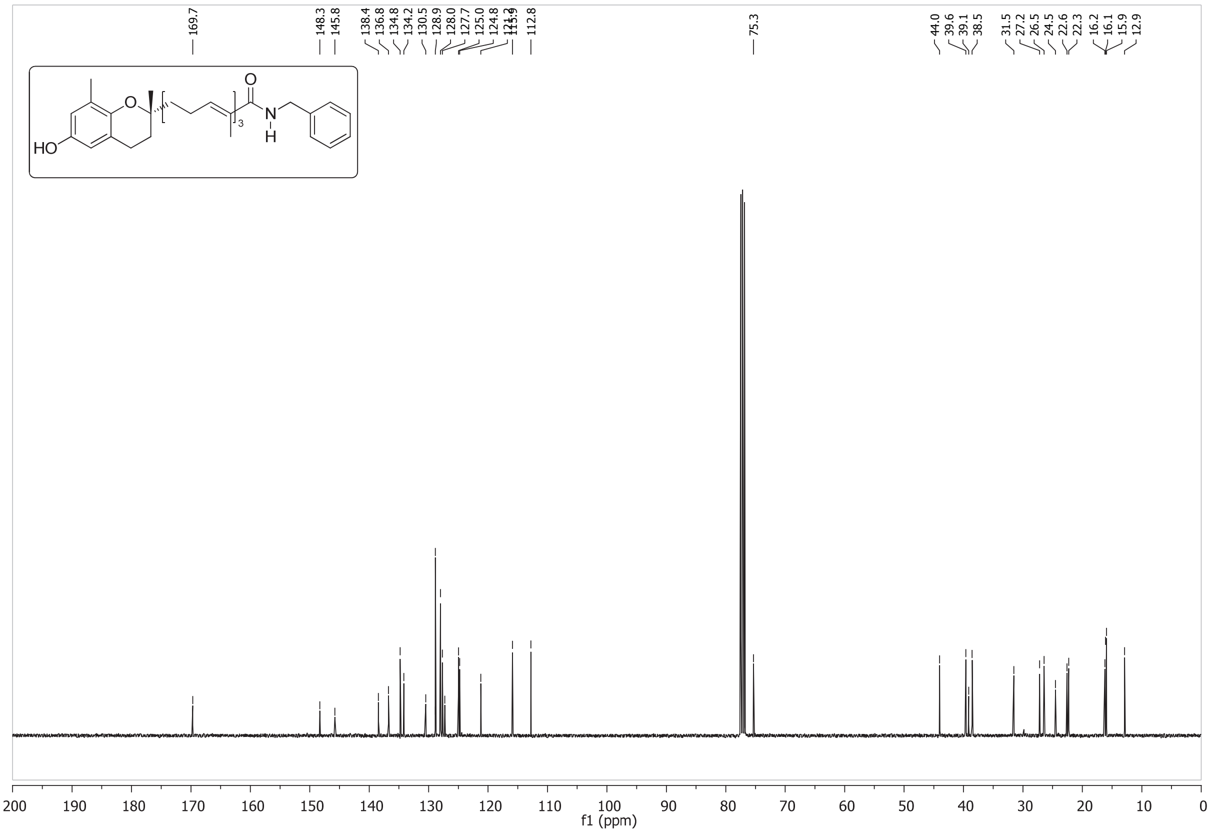

^{1}H and ^{13}C NMR spectra of δ-TE-13'-CONHBz **(7)** in $CDCl_3$

Supplementary references

1. Korb O, Stutzle T, Exner TE. Empirical scoring functions for advanced protein-ligand docking with PLANTS. *J Chem Inf Model* **49**, 84-96 (2009)
